# Bilateral Cross Arm Flaps for Resurfacing Hands After High-Voltage Injury

**Published:** 2019-01-14

**Authors:** Nikhil R. Shah, Wess A. Cohen, Haripriya S. Ayyala, Jonathan Sorkin, David Mathes, Ashley Ignatiuk

**Affiliations:** ^a^Division of Plastic and Reconstructive Surgery, Rutgers New Jersey Medical School, Newark; ^b^Medical Student, University of Colorado, Denver; ^c^Division of Plastic and Reconstructive Surgery, University of Colorado, Denver

**Keywords:** flap, hand, high-voltage, pedicle, resurfacing

## DESCRIPTION

**Figure 1:**
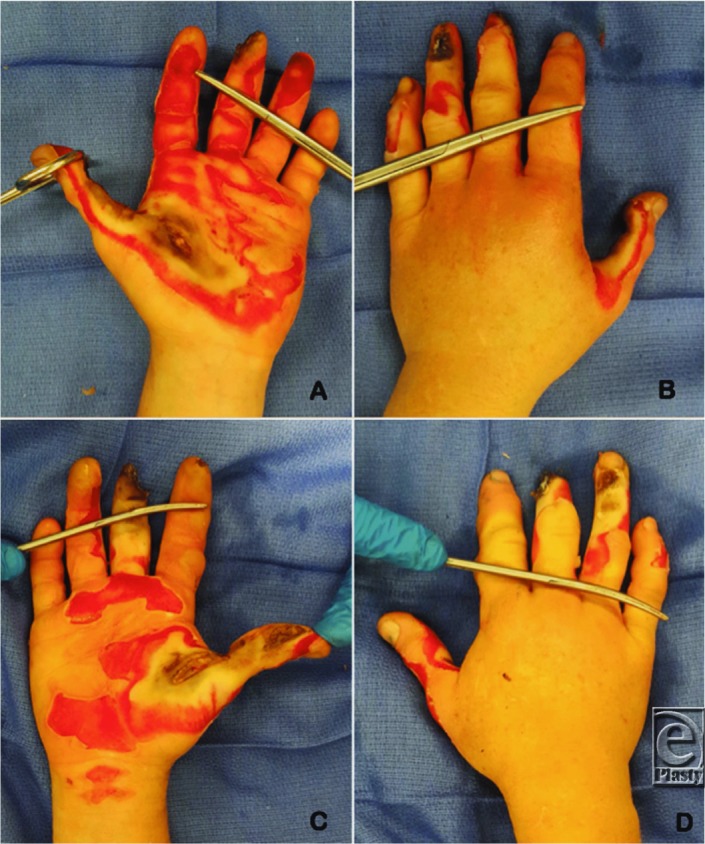
Electrical burns on the hands. (a) Burns seen on the palmar surface of the left hand with severe burns to the thumb, thenar eminence, and second digit. (b) Dorsal surface of the left hand showing severe burns to the thumb and third and fourth digits. (c) Superficial burns seen on the palmar surface of the right hand, with more severe burns across the thumb, thenar eminence, and tip of the third digit. (d) Electrical burns on the dorsal side of the right hand, with more severe burns on the third and fourth digits.

This 43-year-old woman suffered severe bilateral electrical burns to the hands after an accident performing Lichtenberg wood burning—a technique using a high-voltage power supply to run current through a piece of wood producing fractal burn patterns. Electrical burns were present on both hands with the most severe injury being to the left thumb (Fig 1).

## QUESTIONS

1. Why are electrical burns challenging to manage?
2. How can viable tissue and vasculature be identified?
3. What are reconstructive options?
4. What advantages did cross-arm pedicled flaps have for this patient?

## DISCUSSION

High-voltage electrical injuries (>1000 V) carry a high potential for fatality; those who survive present with debilitating damage that hinders future functionality.^1^ Electrical burns differ from chemical and thermal burns in their inherent pathophysiology. These injuries travel beyond skin and subcutaneous tissue, penetrating far into the vasculature, muscles, tendons, and even bones.^2^ As bone has the highest conductance, the initial insult has the potential to spread rapidly and incite destruction of all adjacent tissue. This often precludes the use of local reconstructive options.^3^

**Figure 2:**
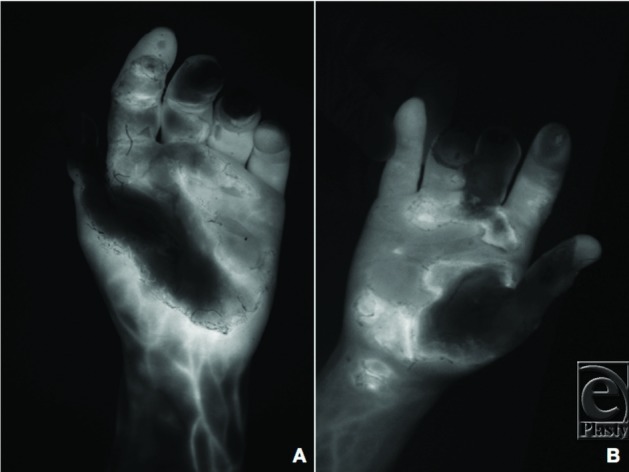
Preoperative fluorescence angiography of left (a) and right (b) hands using indocyanine green dye and a SPY Elite system.

In such cases, the choice of reconstruction is primarily driven by identification of tissue viability. Delays in doing so may increase the chances of perfusion-related amputation.^4^ The sheer nature of injury in the present case made normal visual analysis nearly impossible; thus, indocyanine angiography with the SPY Elite system was used to pre- and intraoperatively overcome poor visibility (Fig 2). This technique has been documented as a method to quickly evaluate tissue perfusion in various de-gloving and crush traumas;^5^^,^^6^ however, to the authors’ knowledge, implementation in the assessment of electrical burns has not been widely discussed.

After debridement of all nonviable tissue and performing amputation of the other digits, the thumbs had full-thickness loss of tissue volarly with exposed interphalangeal joints bilaterally.

Subsequent fusions of the IP joints were required. Salvage of thumb length was the priority and required vascularized tissue transfer. Initially, pedicled groin flaps were proposed for resurfacing, as they have historically seen success in similar situations.^7^^,^^8^ Bilateral application, however, would severely restrict patient mobility and independence in the postoperative period as well as increase risk for immobility-related complications. Consideration of these factors was pivotal, because prior to hospitalization, the patient was a highly active person, frequently participating in rock-climbing, hiking, and exercising in her leisure time. The use of a random pattern pedicle forearm flap was chosen to keep the hands elevated at a comfortable position and allow bilateral shoulder mobility. This would allow the woman to remain active by riding a stationary bike at the hospital.

**Figure 3:**
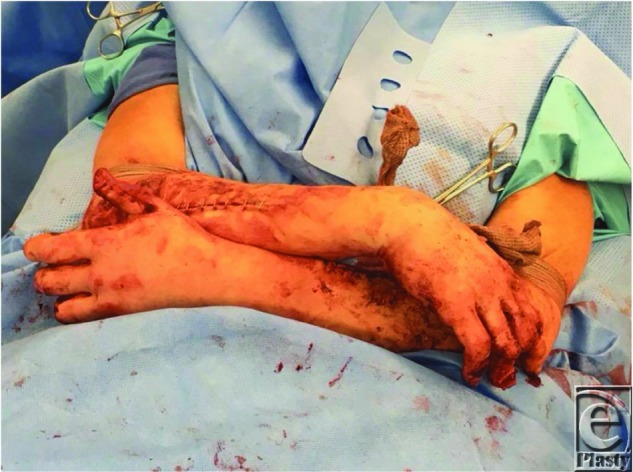
Vascularized flaps sutured to contralateral volar thumbs

**Figure 4:**
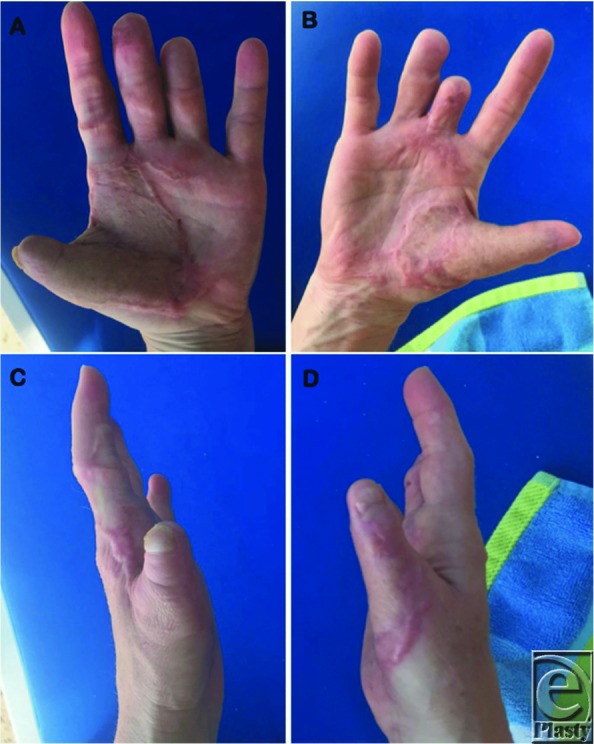
Follow-up photographs 1 year after flap separation showing (a) left palmar, (b) right palmar, (c) left radial, and (d) right radial surfaces of reconstructed thumbs and hands.

**Figure 5:**
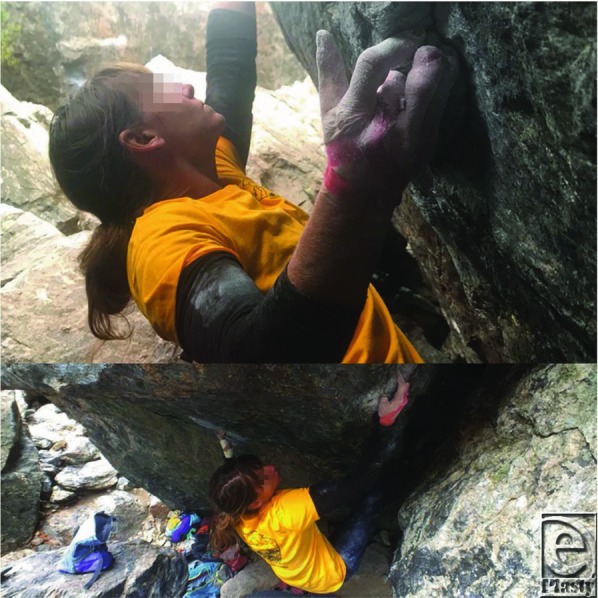
One year postoperatively, the patient was able to return to her active lifestyle, including rock-climbing.

Arthrodesis was performed with a cerclage wire and the arms were crossed to design bilateral cross arm flaps from the dorsal forearm over the mobile. The flaps measured approximately 4 cm × 12 cm bilaterally and were elevated as half ellipses with anterograde flow in the suprafascial plane. The blood supply pattern of the flap was random since an axial vessel was not available and basing the flaps on perforators might have resulted in an unnatural positioning of the patient. After elevation and confirmation of adequate perfusion, the flaps were folded back on themselves and sutured to the contralateral volar thumbs using 3-0 nylon suture (Fig 3). Coban was used to wrap the patient's arms together to relieve tension on the flaps. The division and final inset was performed 3 weeks later. No thinning of the flaps was required and both flaps showed 100% survival. At 6 months, an additional first dorsal metacarpal artery flap was performed to provide sensation to the left thumb tip. Satisfactory results were achieved 1 year postoperatively (Fig 4) and she was able to return to her active lifestyle, including rock climbing (Fig 5).
